# The Role of Adipose Stem Cells in Bone Regeneration and Bone Tissue Engineering

**DOI:** 10.3390/cells10050975

**Published:** 2021-04-21

**Authors:** Wolfgang Mende, Rebekka Götzl, Yusuke Kubo, Thomas Pufe, Tim Ruhl, Justus P. Beier

**Affiliations:** 1 Hand Surgery—Burn Center, Department of Plastic Surgery, RWTH Aachen University Hospital, 52074 Aachen, Germany; wmende@ukaachen.de (W.M.); rgoetzl@ukaachen.de (R.G.); truhl@ukaachen.de (T.R.); 2 Department of Anatomy and Cell Biology, RWTH Aachen University Hospital, 52074 Aachen, Germany; ykubo@ukaachen.de (Y.K.); tpufe@ukaachen.de (T.P.)

**Keywords:** mesenchymal stem cells, regenerative medicine, fracture healing, osteogenic differentiation, osteogenesis, mechanic stimuli

## Abstract

Bone regeneration is a complex process that is influenced by tissue interactions, inflammatory responses, and progenitor cells. Diseases, lifestyle, or multiple trauma can disturb fracture healing, which might result in prolonged healing duration or even failure. The current gold standard therapy in these cases are bone grafts. However, they are associated with several disadvantages, e.g., donor site morbidity and availability of appropriate material. Bone tissue engineering has been proposed as a promising alternative. The success of bone-tissue engineering depends on the administered cells, osteogenic differentiation, and secretome. Different stem cell types offer advantages and drawbacks in this field, while adipose-derived stem or stromal cells (ASCs) are in particular promising. They show high osteogenic potential, osteoinductive ability, and immunomodulation properties. Furthermore, they can be harvested through a noninvasive process in high numbers. ASCs can be induced into osteogenic lineage through bioactive molecules, i.e., growth factors and cytokines. Moreover, their secretome, in particular extracellular vesicles, has been linked to fracture healing. The aim of this review is a comprehensive overview of ASCs for bone regeneration and bone tissue engineering.

## 1. Introduction

Bone regeneration is a sophisticated process influenced by a variety of factors. In young healthy patients, bone tissue shows high self-repair abilities. However, systematic factors, such as an increased age, disease or obesity can negatively affect bone regeneration [1,2,3]. Large defects due to heavy trauma, multiple fracture, infection, or tumor resection, are also disruptive for proper tissue healing [4,5]. Notably, 5–10% of all fracture healing is disturbed, might take months longer or is even impossible [2,6].

The current gold standard for bone defect reconstruction is bone grafting where autologous bone tissue is transplanted to bridge the gap in the bone defect zone. The graft structure is similar to the original bone, which enables growth and regeneration. However, there are limitations to this therapy, such as donor site morbidity and availability of suitable autologous material [5].

A promising approach is bone tissue engineering, which has been successfully applied in a few clinical trials [7,8,9,10]. One approach is to transplant osteogenically induced stem cells into the bone defect zone, which then support the healing process. Within the fracture zone, the cells undergo further osteogenic differentiation, secretion of osteogenic factors, and recruitment of osteoblast progenitor cells. Stem cells can also be combined with allogeneic, alloplastic or xenogeneic scaffolds. These structures are seeded with the cells and support healing by their osteoinductive and/or osteoconductive properties [11].

Stem cells can be isolated from embryonic, fetal and adult tissue. Alternatively, cells are induced into the pluripotent stem-cell state: induced pluripotent stem (iPS) cells. Embryonic, fetal, or reprogrammed cells are associated with major safety, regulatory and ethical problems [12]. In contrast, adult stem cells can easily be isolated from a variety of tissues including adipose-derived stem or stromal cells (ASCs) from adipose tissue, with high osteoinductive and osteogenic potential [11]. These adult stem cells are called mesenchymal stem cells (MSCs). Beside ASCs, bone marrow mesenchymal stem cells (bmMSCs) are another type of MSCs, which also show the typical characteristics of all MSCs. Bone marrow biopsy allows isolation of bmMSCs, which is a procedure with risk of additional morbidity that provides only a low yield of cells, when compared to the surgical procedure for harvesting ASCs: ASCs can be easily harvested through noninvasive procedure and have a significantly higher yield of cells than that obtained for bmMSCs [13]. Moreover, ASCs have a higher proliferation capacity and more colony-forming units compared to bmMSCs [14,15]. Cell therapy requires high numbers of cells for successful application. This could require artificial cell expansion to reach sufficient numbers [16]. However, cell culture increases senescence with every passage, with the consequence of reduced proliferation, changes in morphology, which both could influence the cell function. bmMSCs are more susceptible to senescence and have a shorter life span then ASCs [14,15].

Bone tissue engineering is a sophisticated process, in which there is an interplay between stem cell properties, osteogenic pathways and secretome. Here we review these concepts.

## 2. Bone Regeneration

Bone tissue is able to remodel and self-renew, which happens throughout a human’s lifespan. Their extra-cellular matrix, which consists of water, minerals (e.g., hydroxyapatite, calcium fluoride, and calcium carbonate), and proteins (mostly type I collagen) [17], undergoes constant remodeling. Equilibrium is obtained by synthesizing osteoblasts and degradation from osteoclasts [3]. bmMSCs influence this through their secretome and their ability to develop into osteoblasts [3,18].

### 2.1. Fracture Healing

Fracture healing can take place in two ways: primary healing requires that the fragments are in close contact and immobilized. This happens when a fracture is immediately treated after trauma. A small amount of granular tissue and callus forms between both fracture ends. The cutting cone, which consists of osteoclasts, creates zones between both ends, while osteoblasts interconnect these zones. The new bone is then formed and the fracture is closed [2,19].

However, most fractures close through secondary healing, also called endochondral ossification. This process is divided into four stages: hematoma formation (days 1–5), soft callus formation (days 5–11), hard callus formation (days 11–28), and bone remodeling (day 28–months later) [2].

Trauma causes the fracture itself and additionally leads to rupture of blood vessels inside the bone, which creates a hematoma. The hematoma creates a clot inside the fracture, which induces the recruitment of immune cells, including macrophages, monocytes, and lymphocytes. These cells influence the subsequent process of osteogenesis [20,21]. They initiate and modulate the fracture healing process. In particular, macrophages are crucially involved in bone healing through their secretome [20,22] and by forming a layer above the osteoblast, which is called an osteomac [23]. The macrophages have different phenotypes, such as M1 (the so-called activated or proinflammatory phenotype) and M2 (the alternatively activated or anti-inflammatory phenotype), which are induced during different phases and, in turn, influence different processes of bone healing. The exact mechanisms and participations during bone tissue regeneration are not completely understood [24,25]. The M1 phenotype appears to support the inflammatory response and reduces regenerative osteogenic potential. Moreover, M2 phenotypes have pro-osteogenic effects though their secretome. Kang et al. [25] studied healing of rat calvaria defects and show that M2 secretomes support fracture healing. Furthermore, macrophage deficient mice have fewer MSCs in their bones, decrease bone mineralization and longer fracture healing time [22]. On the other hand, a high and prolonged inflammatory intensity impairs, or even completely inhibits, the tissue healing process [3].

In the second step, granulation tissue, which is rich in fibroin, develops inside the fracture. The growth factors secreted by immune cells include vascular endothelial growth factor (VEGF), which induces vascularization and the outgrowth of blood vessels. Moreover, MSCs are recruited to the fracture site, and they start to proliferate and differentiate into chondroblasts, osteoblasts, and fibroblasts. Chondroblasts help with the creation of a soft cartilage callus inside the fracture [2,19].

The soft cartilage callus is incrementally replaced by a hard bone callus during the subsequent days. This is accomplished by the collaboration of osteoblasts, osteoclasts, chondroclasts and chondroblasts. Vascularization also occurs deeper into the callus, thus facilitating MSCs to invaginate. This process, in turn, fosters the creation of a hard callus. Osteo-progenitor cells start the creation of woven bone from periosteal. At the end of this stage, the callus is completely replaced by bone tissue [2,19].

To complete the healing, the bone must be remodeled, which is achieved through the equilibrium of osteoclast resorption and osteoblast rebuilding. The remodeling aims to create compact bone at the center and lamellar bone at the edge. This process can take months to complete [2,3,20].

### 2.2. Impaired Bone Healing

Fracture healing in 5–10% of patients can fail or be delayed for months [2,6], and this can either be caused by systemic risk factors, such as obesity, malnutrition, smoking, anemia, endocrine conditions, disease and aging [1,2,3] and/or local risk factors extensive fractures from massive trauma, multiple and open fractures, radiotherapy or infection [5].

Older people suffer from reduced bone mass and thus experience more frequent and severe bone fractures [3,26]. Moreover, their MSCs have a reduced commitment to osteogenic lineage and are primed for adipogenesis [27]. Studies have shown that several osteogenic transcription factors are reduced in older MSCs, including MAF bZIP transcription factor [28], Forkhead box P1 [29], and Core-binding factor β [30]. Accordingly, fracture healing is impaired among the elderly, and recovery takes longer. In animal studies, older rats show less bone regeneration, reduced vascularization of their callus, less cartilage, and decreased ossification [31].

Diseases, such as diabetes type I and type II, osteoporosis, and osteogenesis imperfecta, are also a major factor in interrupted fracture healing [3]. Osteoporosis leads to less Ca^2+^ deposition in the bones, making the structure weaker. As such, trauma can result in larger fractures, which often require additional treatment [32]. The impact of osteoporosis on fracture healing has been disputed in conflicting studies [33,34].

Most cases of osteogenesis imperfecta are due to genetic mutation in collagen type I. The disease is associated with more brittle bones and more fractures. The healing of these fractures in many cases results in non-union, which often requires a longer healing time [3,35,36].

Patients with diabetes generally show reduced bone regeneration. In 87% of cases, fractures need a longer healing period. These patients have a 3.4 times higher risk for complications [37]. Thereby, myostatin regulates bone formation, and blocking myostatin improves fracture healing in patients with type 2 diabetes [38]. Furthermore, diabetes is associated with advanced glycation end-products, which are proteins linked to aldose. These proteins can bind to the receptor of advanced glycation end products (RAGE) and lead to a proinflammatory response. They are also linked to an increased number of osteoclasts [37,39], reduce osteoblast ability for bone repair, and decreasing bone mineralization [40].

Moreover, fracture healing can be impaired through infections. *Staphylococcus aureus* is a common pathogen in healthcare settings and is often associated with soft tissue complications [41,42]. The pathogen is also responsible for 30–42% of all infections during bone healing, which can appear because of an open fracture, or bone fixation. Thereby, bone regeneration is disturbed, and antimicrobial therapy is necessary [43].

In an aging society where there is an increase in lifestyle diseases, such as diabetes, it is crucial to have efficient treatment for delayed or failed fracture healing. The method currently used is bone grafting.

### 2.3. Interaction of Inflammatory and Bone Cells during the Fracture Healing and Bone Re-Generation—The Nrf2–Keap1 System

In the mechanism of intractable fractures, oxidative stress is considered one of the main factors that interfere with fracture healing. Oxidative stress is generally caused by an imbalance between oxidation and reduction. During the early phase of fracture healing, reactive oxygen species (ROS) are generated under inflammatory and ischemic conditions [44]. However, the influence of ROS can be normally restricted by protective antioxidant enzymes, capable of stabilizing or deactivating free radicals before cellular components are attacked [45,46,47]. On the other hand, excessive oxidative stress potentially can occur after the fracture in patients with underlying diseases that inherently expose to oxidative stress, as well as disruptive or compound fractures [45]. Excessive ROS can lead to chronic inflammation [48], decrease in osteoblast function and differentiation [49,50], whereas they can activate bone resorption through elevating osteoclast differentiation and function [51]. Thus, these modifications of bone metabolism by oxidative stress affect bone remodeling and regeneration [52].

The Nuclear factor erythroid 2–related factor 2 (Nrf2)-Kelch-like erythroid cell-derived protein with cap ‘n’ collar homology-associated protein 1 (Keap1) system plays an important role in the regulation of the biological response to oxidative stress. In the basal condition, Nrf2 is regulated by the stress sensor Keap1. Under conditions of oxidative stress, stabilized Nrf2 is translocated into the nucleus where it binds to antioxidant-response elements (ARE) in the promoter regions of target genes, resulting in the activation of a variety of antioxidant genes [53,54]. Recently, attention has focused on the role of Nrf2 in fracture healing process. A previous report showed that Nrf2 deficiency decreased fracture callus by using *Nrf2*-knockout (KO) mice [55]. Moreover, Nrf2 can be involved in the control of excessive inflammatory responses [56], the promotion of osteogenesis in MSCs, and angiogenesis through VEGF expression [57], in early phase of fracture healing. In the remodeling phase, Nrf2 also regulates the balance of bone metabolism by suppressing oxidative stress-induced osteoclastogenesis [58]. In light of bone tissue engineering, it is expected that Nrf2 would be a future therapeutic strategy for fracture healing or bone regeneration in patients with intrinsic oxidative stress such as diabetes type II or osteoporosis [45].

## 3. Bone Grafts

**Figure 1:**
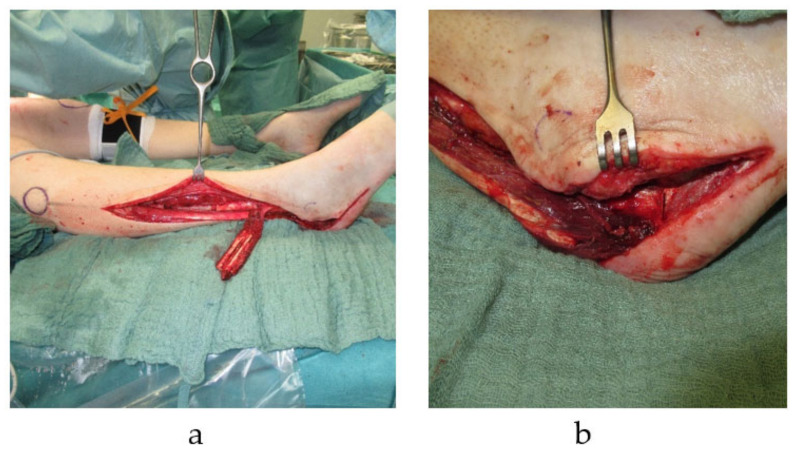
Pedicled autologous bone graft transplantation. Distally pedicled peroneus brevis muscle flap with a fibula segment is harvested from the right lower leg. The transplant is used for reconstruction of a calcaneus defects with a soft tissue defect (**a**). Distally pedicled peroneus brevis muscle flap is transferred into right calcaneal bone defect (**b**).

**Figure 2:**
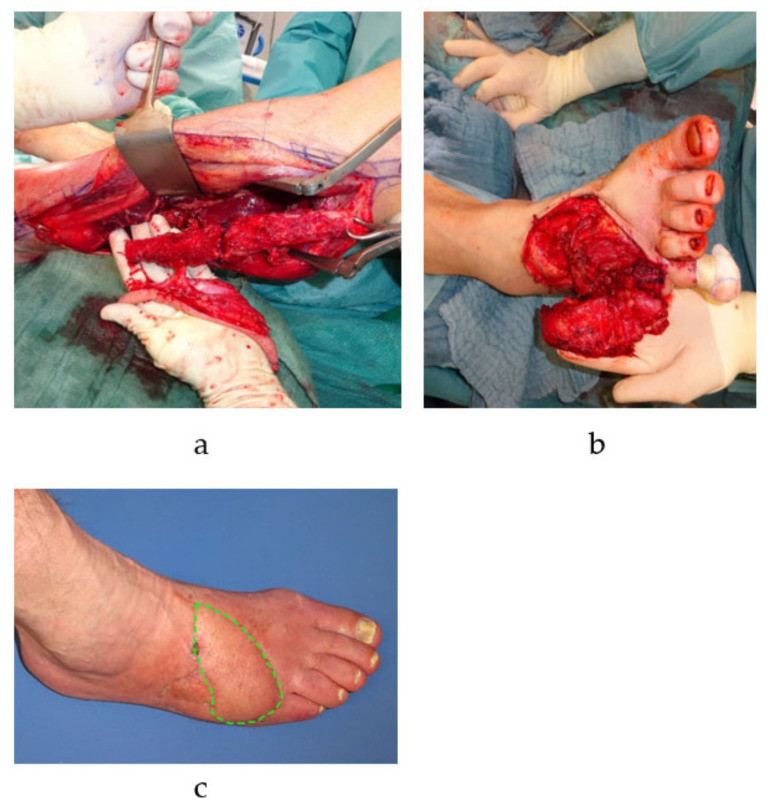
Free microvascular autologous bone transplantation for metatarsal defect. A free fibula transplant with a single-perforator based skin island is harvested from the left lower leg (**a**). The osteocutaneous flap is transplanted into the defect to reconstruct the fifth metatarsal bone of the right foot (vascular anastomosis is performed by connecting the flaps pedicle, i.e., the left fibular artery, to the anterior tibial artery of the right foot in an end-to-side manner) (**b**). The skin island (green dotted line) is twisted 90 degrees and is subsequently used to closes the concomitant soft tissue defect (**c**).

The current gold standard to treat delayed or impaired bone recovery as well as for bone defects is the transplantation of autologous or allogeneic bone grafts [11,20]. Autografts are bone tissues from distant donor sites of the same patient (Figure 1 and Figure 2). They do not induce immunogenic reaction and have osteoinductive effects, e.g., by releasing growth factors. Osteoblast progenitor cells in the graft provide the tissue with osteogenic properties. However, the graft is obtained in another operation, which presents an additional risk of morbidity for the patients. Moreover, it is not always possible to harvest adequate material [20]. Meanwhile, allografts are a decellularized matrix and are obtained from other patients or corpses. Decellularization is accomplished though irradiation or freeze drying [11]. Allografts have a similar bone structure and extracellular matrix as the original bone. Their incorporation is lower compared to autografts [21]; they also present a risk of transmitting infections [59]. Furthermore, they can induce an immune response and can have a high batch variation [20].

Bone grafting is a well-established approach that is used in over 500,000 cases every year in the USA [20,60]. Nevertheless, bone grafts have disadvantages, as described above. In order to avoid such risk factors, alternative approaches are currently under experimental and/or clinical investigation.

## 4. Cell Therapy

Bone tissue engineering is a promising approach for fracture healing, which offers high regeneration capability and biocompatibility. This technique requires stem cells, which can be isolated from various sources [12].

### 4.1. Embryonic- and Induced Pluripotent Stem Cells

Embryonic stem cells (ESCs) are obtained from the inner cell mass of blastocysts. As pluripotent stem cells, they can differentiate into cells of all three germ lines. ESCs have several advantages as a source for bone tissue engineering: self-renewal ability, pluripotency, high genome stability after multiple passaging [61], and sufficient osteogenic potential [62]. However, ESCs availability can be problematic due to low access of available tissue and ethical concerns [12]. Furthermore, undifferentiated ESCs can be carcinogenic and form teratoma, which are tumor tissues with cells from all three lineages [63]. Therefore, ESCs must be cleared of all undifferentiated cells before usage in tissue engineering [13], which is a safety concern.

Overexpression of the genes *Oct4/3*, *Sox2*, *Klf4*, and *c-Myc* in adult cells leads to the creation of iPSC. Shinya Yamanaka et al. [64] were the first group to create iPSC in 2006 from mice fibroblasts. Human iPSCs were created one year later also from fibroblasts [65]. iPSCs are pluripotent cells like ESCs, but they bypass the ethical concerns surrounding ESCs. The osteogenic capacity of iPSCs is similar to that of ESCs [66] and they have been successfully used for bone tissue engineering in animal models [67,68]. The method of creation from iPSCs has given rise to some concerns that they may be carcinogenic [65,69]. Additionally, the transcriptome of iPSCs is similar to cancer cells [12,70]. One method to reduce this is to differentiate iPSCs before usage [71]. During this process, no contamination of undifferentiated cells should remain.

### 4.2. Adult Stem Cells

Friendenstein et al. [72] were the first group in 1970, that showed that adult stem cells have multilineage potential and can be induced into osteogenesis [72]. These cells have immunomodulatory and regenerative capabilities [12]. They are commonly referred to asMSCs, but they were pushed for a name that better reflects their role and origin. Thus, “tissue-specific progenitor cells” and “medicinal signaling cells” have been proposed [73,74]. MSCs are a heterogeneous mix of committed stem- and progenitor cells [75]. The International Society for Cellular Therapy has set four criteria for defining MSCs [76]. These include adherent cells in a standard cell culture, which can differentiate into osteogenic, chondrogenic and adipogenic lineages. Moreover, MSCs must express CD73, CD90 and CD105. Furthermore, they should lack expression of CD11b, CD14, CD19, CD34, CD45, CD79α and the HLA-DR surface marker [76].

MSCs are derived from perivascular niche and can be found in almost all tissues with vascularization [12,77]. Therefore MSCs can be harvested from skeletal muscle [78], umbilical cord [79], skin [80], dental pulp [81], bone marrow [82], and adipose tissue [83,84].

Bone marrow comprises hematopoietic cells, different adipose stem cells, stromal cells and bone marrow MSCs (bmMSCs) [85,86], and many studies have focused on this reliable source of MSCs. bmMSCs have high osteogenic differentiation and proliferation potential and a low risk of immunogenicity. Therefore, bmMSCs have often been used for bone tissue engineering. Li et al. [87] used bmMSCs for the recovery of orbital defects in rats. Jingying et al. [88] repaired calvarial defects in rabbits with bmMSCs. However, the extraction of bone marrow is an invasive procedure and can be dangerous to patients. Moreover, the concentration of cells per mL bone marrow aspirate is only 0.001–0.01% [13], necessitating the intensive use of cell culture and growth factors to obtain sufficient cells for therapeutic approaches, such as bone regeneration. The high heterogeneity of bmMSCs is another problem: bmMSCs become homogenous only after several passages [89]. Heterogeneous bmMSCs have reduced osteogenic differentiation and different morphologies [90,91].

### 4.3. Adipose-Derived Stem or Stromal Cells

In addition to bmMSCs, ASCs are another group of MSCs used in many studies for bone tissue engineering [11]. They are characterized to be positive for the surface markers CD13, CD29, CD44, CD73, CD90 and CD105. Moreover, ASCs should lack CD31, CD45 and CD235a. They can be distinguished from bmMSCs by the markers CD106 and CD36; ASCs are positive for the first one and negative for the latter, vice versa for bmMSC [92]. Both MSCs groups origin from different stem cell “niches”, which might account for the differences in their differentiation potential. Thus, ASCs are more primed to develop into adipocytes, and bmMSCs into ostoblasts, although the osteogenic potential of ASCs and bmMSCs is disputed. The majority of studies found stronger osteogenic capacity in bmMSC [93,94,95,96], but some could not find any difference [14,97,98]. Especially due to the differences in the differentiation potential and also the secretory activity of the cells, the International Society for Cellular Therapy (ISCT) have proposed the nomenclature of ASCs vs. bmMSCs, to highlight the difference between both groups [92].

ASCs can be harvested through liposuction from adipose tissue, a safe and minimally invasive procedure. Moreover, lipoaspirate has a high yield of ASCs at 2% and over 10^7^ ASCs can be isolated from 300 mL lipoaspirate [99,100]. ASCs also provide high osteogenic potential. The secretome has beneficial effects, as it recruits other progenitor cells and can induce osteogenic differentiation [5,101]. Allogenic ASC usage is theoretically possible because they have a low risk of immunogenicity, since ASCs have a low number of MHC class II molecules, immune cell stimulation factors CD80, CD86 and CD40 [102,103], and release an immunosuppressive secretome [13].

ASCs are perivascular progenitor cells [11,104] derived from the stromal vascular fraction (SVF), which additionally consists of endothelial cells, fibroblasts, pericytes, monocyte, macrophages, lymphocytes, blood-derived cells, vascular smooth muscle cells, and preadipocytes [105]. Depending on different factors, including the age of the patient, donor site, and type of fat tissue, the SVF contains different numbers of ASCs [106,107]. Moreover, freshly isolated ASCs can have heterogeneity in their lineage potential. Thus, fresh adherent ASCs are composed of 21% tri-, 31% bi-, 29% unipotent cells and 19% without lineage potential. ASCs become more homogeneous after passaging and reducing the remaining stromal cells [108].

ASCs are often cultivated in a basal medium with 10% fetal calf serum (FCS) [5]. Some researchers add supplements, such as fibroblast growth factor 2 (FGF-2) or transforming growth factor (TGF-β) [109]. As FCS is a xenogeneic substance, autologous serum was proposed as an alternative nutrient source. The nutrient source shows only minor impact on osteogenic differentiation of ASCs [110], which indicates that autologous serum could be a viable alternative. Comparing studies on ASCs can be difficult since protocols are often not standardized and this can influence the properties of the ASCs [111,112].

## 5. Osteogenic Stem Cell Differentiation through Bioactive Factors

### 5.1. ASC Osteogenic Differentiation

ASCs have a multilineage potential of differentiation and can e.g., follow an adipogenic, chondrogenic or osteogenic fate. Each lineage has major regulators that determine the fate of the cells. For osteogenesis, these are Runx2 and Osterix (Sp7 transcription factor) [113,114]. Regulation involves a complex network and several signaling pathways control osteogenic differentiation, among others bone morphogenetic protein (BMP)- [115], Notch- [116], Wnt- [112] and Hedgehog-signaling [117]. The Wnt signaling pathway is key as it serves as governor between ASC lineages. It channels ASCs away from adipogenic or chondrogenic lineage to osteogenic differentiation by increasing Runx2 and Osterix [118].

**Figure 3:**
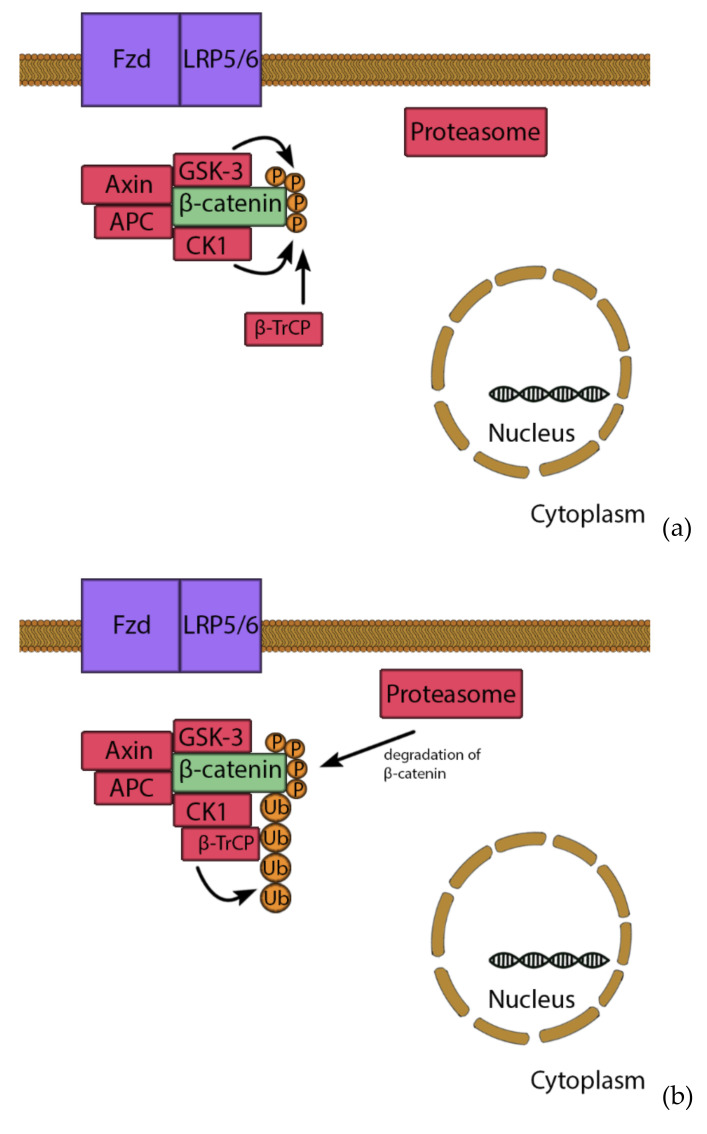

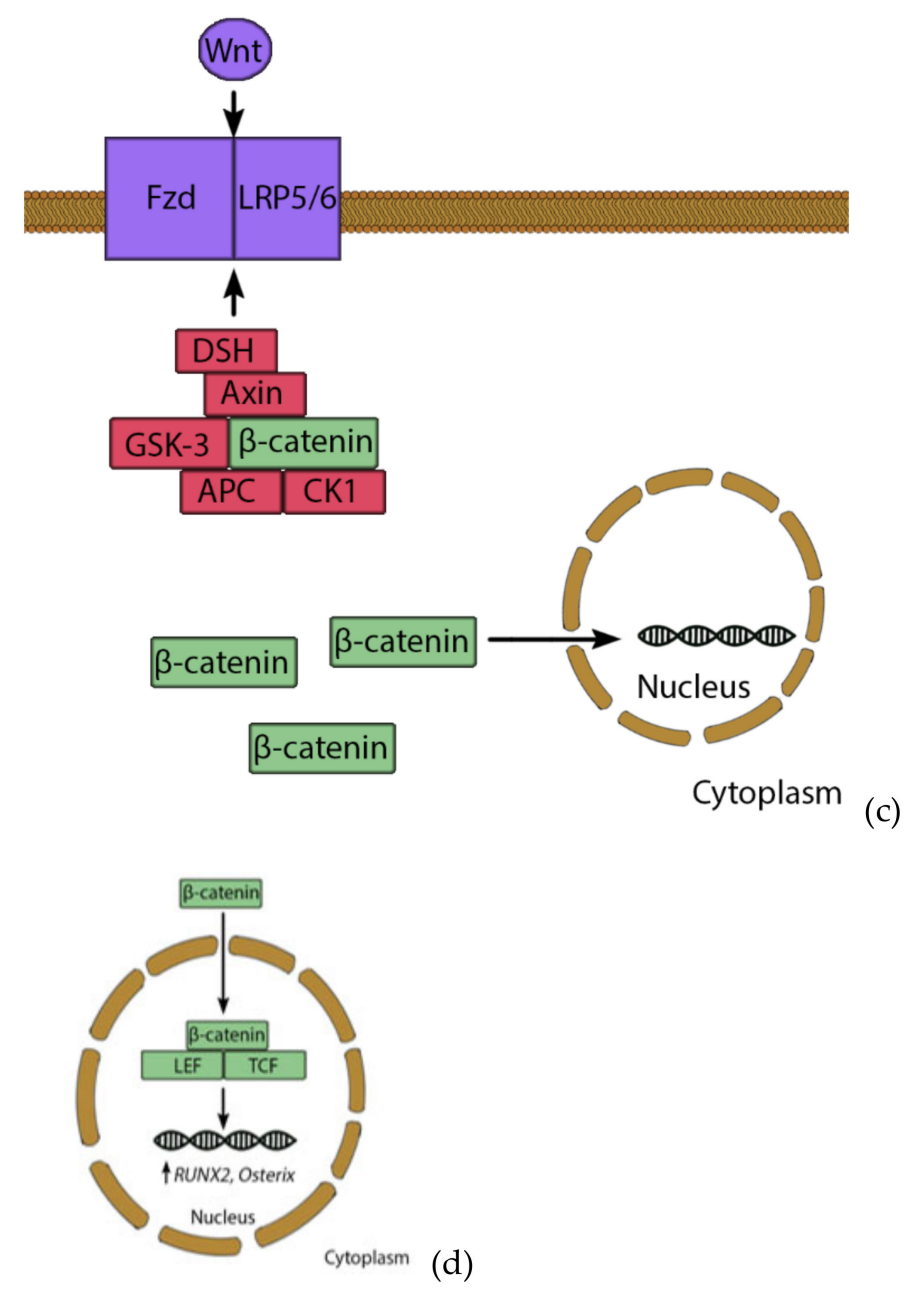
Wnt/β-catenin pathway. Under basal conditions, β-catenin is bound by a removal complex consisting of Axin, GSK-3, APC and CK1 (**a**). GSK-3 and CK-1 marked β-catenin though phosphorylation, which recruits β-TrCP. β-TrCP leads to ubiquitination and degradation of β-catenin (**b**). However, stimulation of Wnt leads to activation of disheveled proteins (DSH), which binds the removal complex to the receptor. Therefore, β-catenin is not degraded and can enter the nucleus (**c**), where it forms a complex with TCF and LEF transcription factors. This complex induces expression of e.g., *RUNX2* and *Osterix*, leading to osteogenic differentiation (**d**).

Wnt signaling acts over two pathways: via a canonical β-catenin pathway (Figure 3) and one via a noncanonical pathway [112,119]. β-catenin is a pro-osteogenic transcription factor. However, unstimulated conditions lead to the phosphorylation of β-catenin by a removal-complex, which leads to the proteolytic depletion of β-catenin. The pathway starts when Wnt binds to the Fizzled receptor, which is supported by low density lipoprotein receptor related protein 5/6 (LRP5/6). The signal leads to the disheveled proteins (DSH) binding to the removal complex and attaching it to the receptor. The complex cannot mark β-catenin for degrading and it moves to the nucleus. There, β-catenin, lymphoid enhancer binding factor 1 and T-cell factor1,3,4 (TCF1,3,4) form a complex, which supports expression of osteogenic genes, such as *Osterix* and *RUNX2* [120]. In the noncanonical pathway, Wnt binds to the receptor tyrosine kinase-like orphan receptor-1 and 2 (ROR1/ROR2), which leads to the activation of a G-protein, whereby the intracellular Ca^2+^ from the endoplasmic reticulum is released [121]. Ca^2+^ acts as second messenger and is very important for several signaling pathways; including osteoblast proliferation [122].

ASCs for bone regeneration therapy can be induced into the osteogenic lineage using a differentiation medium comprising dexamethasone, beta-glycerophosphate and ascorbic acid [123]. Dexamethasone was investigated to decrease necrosis, increase proliferation and stimulate osteogenesis, in particular through the Wnt/β-catenin pathway, which increases among others the osteogenic regulators Runx2 and Osterix. Moreover, dexamethasone upregulates Runx2 activity. Beta-glycerophosphate supports osteogenesis by acting as a phosphate source for several reactions, and ascorbic acid increases production and secretion of pro-collagen [112,123].

### 5.2. Supplementary Substances as Osteogenic Stimuli

Supplementary substances added to the medium can accelerate and/or increase overall potential for osteogenic differentiation. Furthermore, ASCs harvested from patients with systemic conditions such as osteoporosis, or aging, may suffer from a reduced inherent potential for bone tissue engineering [124,125], which makes therapeutic treatment necessary.

**Figure 4:**
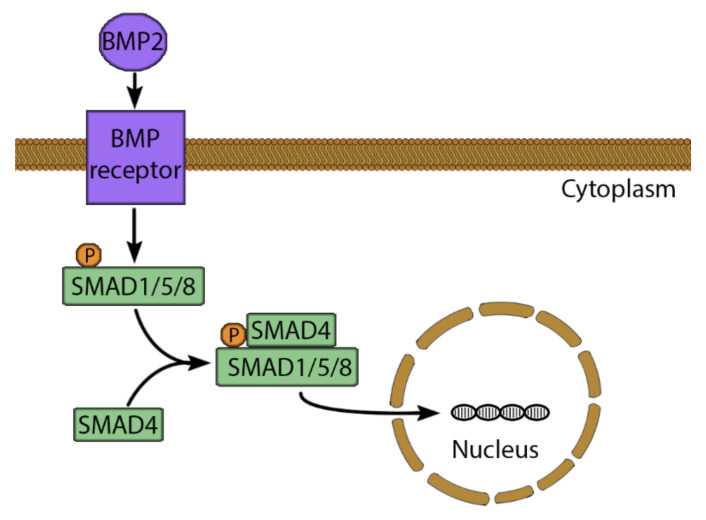
The BMP-2 signaling pathway. BMP-2 binds to a serine/threonine kinase receptor, leading to phosphorylation and activation of SMAD1/5/8. Therefore, SMAD4 can bind to SMAD1/5/8 to form a complex. This complex moves to the nucleus and binds to a specific SMAD binding element. This stimulates the expression of osteogenic genes such as RUNX2 and Osterix.

Growth factors can be added to increase osteogenic potential, proliferation, vascularization, migration and differentiation of progenitor cells [11]. The BMPs are a major pro-osteogenic growth factor group [126]. There are 16 different types of BMPs (BMP-1-16), which belong to the TGF family [127]. The BMP signaling pathway starts by binding to Ser/Thr kinase receptors, which influence the cells via multiple pathways. The most potent induction acts via the Smad transcription factor (Figure 4), whereby binding of BMP-2 or -3 activates the receptor, which phosphorylates the regulatory complex of Smad1/5/8. This allows the attachment of Smad4 to the complex, which moves to the nucleus to increase expression of *RUNX2* and *Osterix* [112,115]. Moreover, BMP influences the cell via the messenger molecules: extracellular signaling-regulated kinase (ERK), c-Jun N-terminal kinase (JNK), p38 and mitogen-activated protein kinase (MAPK), which all influence osteogenesis [112,116]. BMP-2 has been approved by the US Food and Drug Administration for clinical usage in 2003 and has been used successfully in clinical trials for spine fusion and tibial fractures [109,128].

**Figure 5:**
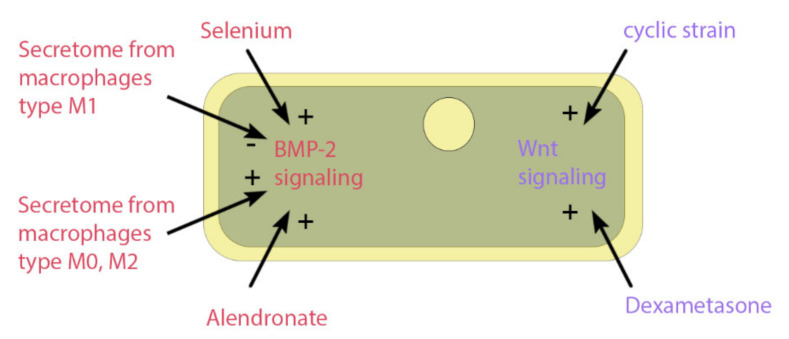
Impact on osteogenic signaling pathways. The figure shows the impact of pharmacological or mechanical stimuli on the osteogenic signaling pathways of BMP-2 or Wnt. The factors include selenium particles, secretome from different phenotypes of macrophages, the drug alendronate, the osteogenic differentiation medium component dexamethasone, and dynamic cell culture through a cyclic strain.

Several studies have used a wide variety of substrates to increase the osteogenic potential of ASCs, such as vitamin D3 [129], alendronate [130], selenium [131], and platelet-rich plasma [132]. Figure 5 shows the influence that substrate or mechanical stimuli have on osteogenic signaling pathways of BMP-2 and Wnt. Other factors involve the inflammatory response, which is the first step of fracture healing (described above). Lipid polysaccharide (LPS) from the membrane of Gram-negative bacteria lead to a proinflammatory immune response and can affect ASCs through the toll-like receptor 4 (TLR4). This receptor activates the NF-κB pathway [133], which influences a variety of cell functions, including proliferation, apoptosis, inflammatory responses, and osteogenic differentiation [134,135]. Peters et al. [136] have shown in in vitro ASCs studies that LPS increases the osteogenic markers of extracellular calcium content and alkaline phosphatase (ALP) activity. Moreover, the endocannabinoid system influences a wide variety of systems in nearly all tissues, including modulation of the inflammatory response, cell stress, wound healing [137,138] and bone formation [139]. In ASC cell culture, some endocannabinoids could improve osteogenic differentiation, including plant-derived cannabidiol (CBD) [140] and endogenous N-arachidonoylethanolamine (AEA) [141]. In addition, the combination of LPS and CBD treatment on ASCs seems beneficial, since LPS alone increases cellular stress, and CBD can stop this [137].

### 5.3. Mechanical Stimuli on Osteogenic Differentiation

Regulation of MSCs during embryonic development and fracture healing is influenced by mechanical stimulation. Extrinsic mechanical forces can be used to mimic these effects and also support osteogenic differentiation of cultured ASCs [142].

One example is tensile strain through uniaxial or equiaxial stretching, where cells are seeded with osteogenic differentiation medium in a chamber made of flexible material, such as polydimethylsiloxane (PDMS). The combination of biochemical and mechanical stimulation increases the osteogenic differentiation, when compared to one factor applied solely. Furthermore, stretching increases focal adhesion and the amount of actin in the cytoskeleton [143,144]. This mechanical stimulus has been shown to increase the Wnt signaling, which induces cellular osteogenic differentiation [112]. Mechanical strain also impacts oxidative stress regulation through the Nrf2/ARE pathway. A study with MSCs from dental pulp shows that cyclic strain increases ROS and the inflammatory response. This activates Nrf2/ARE signaling, increasing the expression of antioxidant enzymes [145,146].

Furthermore, vibration loading is used to create a dynamic input onto ASCs in vitro. In this approach, the cells are seeded on e.g., a petri dishes and loaded onto a platform that can vibrate at specific frequencies. Vibration loading of ASCs results in an increase of osteogenic markers, including ALP activity, collagen levels, and the Ca^2+^ deposition in the ECM. Moreover, vibration loading inhibits adipogenic differentiation [147,148].

Flow intensity over the cells also impacts ASCs, which has been investigated in 2D and 3D culture. Tjabringa et al. [149] studied pulsating fluid flow in 2D; the stimulus was applied for one hour and the gene expression of *RUNX2* levels increased while *secreted phosphoprotein 1* (*SPP1*) (gen of osteopontin) stayed unaffected. Runx2 is associated with the early phase of osteogenesis and osteopontin with later stages. Therefore, it has been proposed that a pulsating fluid flow may induce the early stages of osteogenesis [150]. Fröhlich et al. [151] seeded ASCs on a decellularized bone graft and used fluid flow in a bioreactor to support osteogenesis. Perfusion increase mineralization compared to motionless culture after 5 weeks. However, in 3D culture, it is unclear whether the effect is a result of the mechanical stimuli or the better cell culture conditions (e.g., gas- and nutrition exchange and dispensation of cells) [142].

Electromagnetic fields that were applied as a direct or alternating current between 2 and 123 Hz affect osteogenesis of ASCs. Runx2, ALP activity, collagen I, and osteopotin increased [112,152]. The electric signaling is associated with intracellular Ca^2+^ release from the endoplasmic reticulum, which is of relevance for several physiologic processes, including osteogenesis [112].

## 6. Secretome and ASCs in Crosstalk during Bone Regeneration

### 6.1. The ASC Secretome

Fracture healing is a complex process, which is influenced by the crosstalk of surrounding tissues and related wound factors. Seeding ASCs in the fracture support bone recovery. They exert paracrine effects through the release of soluble factors (e.g., cytokines and growth factors) and small extracellular vesicles (EVs). The ASC secretome influences the surrounding tissue in multiple ways. It supports angiogenic, osteogenic differentiation and progenitor cells are recruited to the fracture site. Furthermore, ASCs have immunomodulation properties [5,101]. It has been shown that they interact with the innate immune system and reduce the number of B-cells in a fracture [153].

### 6.2. Extracellular Vesicle from ASCs

EVs also influence stem and progenitor cells in the tissue crosstalk. They can be found in nearly all tissues and have received more attention in recent years. The vesicles consist of a lipid bilayer surrounding the loaded cargo. They regulate many processes and often have pleiotropic effects because they are very heterogeneous in nature [154]. EVs are involved in the horizontal transfer of mRNA, miRNA, other noncoding RNA, proteins, lipids [155], and mitochondrial DNA [156]. For cell therapy and tissue engineering, two types of EVs are important: microvesicles and exosomes. Compared to exosomes, microvesicles are larger, measuring 100–1000 nm. They are created through an outward budding of the cellular membrane, which is regulated by small GTPase [157]. On their surface, microvesicles contain selectine and integrine as markers [158]. Exosomes are 30–100 nm in size and are created at the endosome. They bud from the endosomal membrane into the lumen, which then creates the multivesicular endosome. EVs are released when the multivesicular endosome fuses with the cellular membrane [159]. Various markers, such as CD9, CD63, CD81, HSC70, ALIX, and flotillin-1 can be used to detect exosomes [160]. EVs influence tissue crosstalk through surface markers, such as membrane molecules [161] and their cargo-like regulatory RNA and factors of signal pathways [158]. It has been shown that EVs can act directly and bind to specific cells [162].

EVs are relevant to bone regeneration. In one study, Li et al. [163] found that embedded EVs from ASCs in a scaffold increase healing after six weeks. This study was conducted in a mouse model with a calvarial defect. EVs have osteogenic effects through influencing the phosphatidylinositol 3-kinase/protein kinase B (PI3K/AKT) signaling pathway [164] and the miRNA196a [165]. Moreover, they are involved in the recruitment of MSCs toward a fracture [166,167]. EVs have proangiogenic effect [168] and pro/anti-inflammatory effects [169]. However, the regulatory influence of EVs is not fully understood; it seems to depend on specific tissues and is very dependent on other factors [170]. For example, Zhu et al. [171] found that diabetes type 1 reduces the osteogenic effects of EVs.

## 7. Preclinical Application

In vitro studies facilitate a deeper understanding of the fundamental mechanics of cells, such as signaling pathways and possible inducing and/or supportive stimuli. However, the systematic interplay of various factors necessitates in vivo studies to acquire comprehensive results regarding the use of ASCs for bone tissue engineering [5,172].

ASCs can be directly injected into a fracture site or seeded onto an appropriate scaffold, which provides mechanical stability and protection for the cells. Furthermore, it can mimic the biophysical signals from the extracellular matrix, which can support osteogenic differentiation. The scaffold offers binding sites for the cells and space for calcium deposits. Thus, the size and connectivity of pores play an important role for artificial bone tissue engineering. The choice of pore size and stiffness of the structure is a compromise. Wider pores allow the migration of cells, greater secretion of bone material, and easy vascularization. However, they also reduce the surface for cell attachment. Thus, a wide variation in pore size is possible, depending on the scaffold material [11,109]. Besides pore size, integrating blood vessels into the scaffold is another strategy to increase vascularization. The arteriovenous loop (AV-loop) model is such a strategy and was shown to improve intrinsic vascularization [173,174,175].

A range of materials is available for the bone tissue engineering scaffolds, which are revised in [11], including ceramide, e.g. hydroxyapatite (HA), coralline-derived hydroxyapatite (cHA), β-tricalcium phosphate (β-TCP) [173,176,177], bioglasses [178], synthetic or biological polymers, e.g. collagen, fibrin [179], fibronectin [180], polylactic acid (PLA) [180], and poly lactic-co-glycolic acid (PLGA) [130,181] and a combination of multiple materials [182,183]. Combinations of different materials can support osteogenic differentiation. An example is PLA scaffolds mixed with mineral substrates of dicalcium phosphate dihydrate or hydraulic calcium silicate [184].

Numerous preclinical studies have examined ASC for bone tissue engineering (Table 1). These studies should show which additional factor and scaffolds can be transferred to a in vivo model. The goal would be to reduce the necessity for additional factors and still provide sufficient osteogenic potential. This would facilitate the clinical application. Today, US Food and Drug Administration permits the addition of the growth factors BMP-2 and PDGF-BB (platelet-derived growth factor BB) as bioactive molecules for bone tissue engineering [128,185,186].

Comparability of the studies is difficult since various markers, measurements and time points have been used. Therefore, it would be helpful to utilize more uniform protocols. The ISCT has proposed that osteogenesis of ASCs could be measured by activity and/or expression of specific markers like alkaline phosphatase, osterix, osteocalcin, runx2 and bone sialoprotein [92].

Penington et al. [180] compared undifferentiated ASCs vs. ASCs in osteogenic lineage for the recovery of a skull defect in rabbits. Both cell types were seeded on a PLA/fibronectin scaffold and osteogenically determined ASCs demonstrate greater bone regeneration at six weeks after implantation. Ko et al. [181] studied a new method of immobilized growth factors in scaffolds, which then were gradually released. Therefore, a layer of dopamine was placed on a PLGA scaffold and then BMP-2 was attached via a catechol reaction. This method was applied to a calvarial defect and increased the osteogenic differentiation of ASCs when introduced inside a bone defect. Zhang et al. [187] modified ASCs with vectors to increase *bFGF* expression and injected them into femoral fractures of mice. These transfections led to an increase in growth factor secretion (VEGF), angiogenesis, callus mineralization, and bone formation. Wang et al. [130] treated a calvarial defect in rats with ASCs seeded on a PLGA scaffold and alendronate. Alendronate was injected into the injury and increased bone regeneration after 8–12 weeks. Deng et al. [177] transfected ASCs with an *miRNA-31* lentiviral vector. A rat calvarial defect was treated with a β-TCP scaffold seeded with these cells. The transfected cells increased healing, bone volume, and mineralization.

## 8. Discussion

Bone regeneration after fracture can be delayed or even fail due to age, disease, or extensive damage [2]. This is characterized by a deficit of bone remodeling and regeneration. It is often associated with a lack of stem cell recruitment, slow proliferation, low growth factor levels, and a prolonged inflammatory response [193].

Therefore, ASC-based cell therapy could be a new treatment. Currently, the topic is attracting much interest, including many publications and clinical trials. A search of clinical trials on www.clinicaltrials.gov (accessed on 17.03.2021) for adipose stem cells and bone shows 22 results. In this broad discussion, our goal was to show the interplay of the cellular and molecular properties of ASCs as tools for bone regeneration. We had a special emphasis on the in vivo translation. Thereby, we aim to show that ASC-based therapy is promising and could replace the current gold standard of bone grafts [20].

For treatment, adipose tissue is harvested through a noninvasive method. ASCs are isolated and receive optional osteogenic stimulation. Afterwards, they are implanted into the fracture. The role of ASCs is to support bone regeneration. They have osteogenic differentiation potential, immunomodulation abilities and can recruit further MSCs towards the fracture. Moreover, the ASCs’ secretome facilitates bone regeneration. It contains growth factors, cytokines and EVs. These substrates include proangiogenic (e.g., VEGF) and pro-osteogenic (e.g., BMP-2) factors [5,158]. This shows that ASCs are a promising tool to deal with a prolonged or failed bone regeneration process, which has been applied in clinical use [7,8,9,10]. However, ASCs are far from an established method in clinical routine.

Osteogenic differentiation is a complex system involving multiple pathways. The secretome of ASCs and surrounding bone tissues significantly impact differentiation, and further studies are necessary to fully understand their mutual influence. Additionally, new ways to increase the osteogenic potential of ASCs could be beneficial for treatment. In our opinion, scaffolds coated with growth factors with a controlled release of these factors are particularly interesting [181]. They allow the local administration of osteogenic factors over time. This could help reducing side effects caused by systematic application and would promote the approval of addition factors for bone tissue engineering. Further studies can help bring bone tissue engineering into clinical daily routine.

## Figures and Tables

**Table 1:** Preclinical studies of ASCs as tool for bone tissue engineering.

Experimental Model	Animal	Scaffold	Addition	Reference
Calvarial defect	Rat	PLGA	Alendronate	[130]
Calvarial defect	Mouse	HA/PLGA	rhBMP-2	[183]
Calvarial defect	Rat	β-TCP	*miRNA-31* vector	[177]
Calvarial defect	Mouse	PLGA	Scaffold coated with polydopamine and immobilized rhBMP-2	[181]
Calvarial defect	Mouse	PLGA	*BMP-2* and *miR-148b* vector	[188]
Calvarial defect	Rat	PLGA	Low power laser irradiation	[189]
Skull defect	Rabbit	PLA	Scaffold coated fibronectin	[180]
Ulnar defect	Minipigs	Decellular bone graft	*rhBMP-2* and *rhVEGF* vector	[190]
Vertebral bone void defect	Rat	Fibrin gel	*rhBMP-6* vector	[191]
Femoral defect	Rat	β-TCP	*BMP-2* and *BMP-7* vector	[192]
Femoral defect	Mouse	-	*bFGF* vector	[187]
AV-loop	Rat	HA granules	Combination of ASCs and human umbilical vein endothelial cells	[173]

## Data Availability

Not applicable.
